# Analysis of Sodium Content in 4082 Kinds of Commercial Foods in China

**DOI:** 10.3390/nu14142908

**Published:** 2022-07-15

**Authors:** Zhilin Hao, Li Liang, Dandan Pu, Yuyu Zhang

**Affiliations:** Beijing Key Laboratory of Flavor Chemistry, Beijing Technology and Business University (BTBU), Beijing 100048, China; hzl15716324037@163.com (Z.H.); gcfll@126.com (L.L.); 18518351472@163.com (D.P.)

**Keywords:** commercial foods, pre-packaged foods, sodium content, nutrition label, salt reduction, healthy diet

## Abstract

High-sodium intake is associated with the increased risk of hypertension and cardiovascular disease. Monitoring and analyzing the sodium content in commercial food is instructive for reducing sodium intake in the general population. The sodium content of 4082 commercial foods across 12 food groups and 41 food categories was collected and analyzed, including 4030 pre-packaged foods and 52 artisanal foods. The food group with the highest average sodium content (6888.6 mg/100 g) contained sauces, dressings, springs and dips, followed by bean products (1326.1 mg/100 g) and fish, meat and egg products (1302.1 mg/100 g). The average sodium content of all the collected commercial foods was 1018.6 mg/100 g. Meanwhile, the sodium content of non-alcoholic beverages (49.7 mg/100 g), confectionery (111.8 mg/100 g) and dairy products (164.1 mg/100 g) was much lower than the average sodium content of the 12 food groups. The sodium contents of different food groups and categories were significantly different. The proportion of high-sodium food (600 mg/100 g) was more than one-third of all the products. There are a few products marked with salt reduction on the package. Sixteen salt-reduced products were collected, which belong to the food category of soy sauce and account for 16% of all the soy sauce products. The average sodium content in salt-reduced soy sauce is 2022.8 mg/100 g lower than that of non-salt-reduced soy sauce products. These data provide a primary assessment with sodium content in commercial foods and potential improvements for the food industry to achievement the goal of sodium reduction.

## 1. Introduction

Sodium chloride, known as common salt, is widely used as an additive in food processing to improve food flavor, texture, and food preservation [1]. Sodium-ion is a constituent in cellular fluids, and it is indispensable for muscle contraction, the transmission of nerve impulses, and regulation of osmotic and blood pressure [2,3,4]. However, excessive sodium intake is associated with the increased risk of diseases such as high blood pressure, cardiovascular disease and gastric cancer [5,6]. The effect of dietary sodium on blood pressure varies from person to person because of their different salt sensitivity. Excess salt could be excreted by the kidneys or sweat when salt intake increases. However, salt-sensitive individuals have defects in this mechanism, and the excessive salt is accumulated in the body and manifests as hypertension [7]. About 90% of the ingested sodium comes from salt added during production, cooking or table salt [8]. Moreover, about 77% of sodium intake is from commercial foods and meals in restaurants [9,10]. To prevent and control cardiovascular diseases and diseases related to a high-sodium diet, the World Health Organization (WHO) recommended that sodium intake should be less than 2000 mg per person per day for adults, but few countries have yet to achieve this goal [11,12]. Additionally, the WHO and World Health Assembly recommend a 30% reduction in population sodium intake by 2025 [13]. However, the intake of sodium chloride (NaCl) in China is more than twice the daily limit (5 g) recommended by the WHO [14]. Moreover, China has the highest mortality rate from diseases related to a high-sodium diet in the world [15].

To reduce the sodium consumption, multiple approaches and policies have been implemented worldwide. Since 2003, the UK has introduced a series of salt reduction policies, including public awareness campaigns, food labeling and voluntary reformulation of foods. From 2001 to 2011, the average salt intake in the UK fell from 9.5 g/day to 8.1 g/day [16]. The South African government introduced legislation in 2013 to limit the maximum sodium content in the processed food category. In the early days of the policy, most of the targeted foods in South Africa had already reached the 2016 maximum sodium limit [17]. In China, it is recommended that the daily salt intake for an adult should not exceed 6 g in the Chinese Dietary Guidelines (2016). The Chinese Government has issued a series of related policies, such as the National Nutrition Plan (2017–2030), the “Healthy China 2030” Plan and other related policies, which propose to achieve the goal of reducing the national per capita daily salt intake by 20%.

Nutrition labels can provide nutrition information on pre-packaged foods, retain freedom of choice, and reduce the cost of information search [18]. In addition, marking the sodium and/or salt content on the nutrition information panel of pre-packaged foods also contributes to the reduction in salt [19]. Regarding the sodium content on the nutrition label, many countries divide the sodium content into different levels and distinguish them according to different colors to help consumers choose healthier foods [20]. For example, the UK has marked the salt content on food labels and indicated the recommended intake. In food outsourcing, green, amber and red are used to make eye-catching indications of low-, medium-, and high-salt content [21]. The Chinese Government introduced nutrition labeling regulations (General Rules for Nutrition Labelling of Prepackaged foods-GB7718-2011) in 2011 and formally implemented them on 1 January 2013 [22]. The rules compulsorily regulated that the nutrition label of pre-packaged foods must contain information such as energy value and the content of fat, protein, carbohydrate and sodium. The accuracy of the information on nutrition labels impacts efforts to monitor sodium content in foods and reduce dietary sodium intake [23]. There are few reports on the summary and analysis of sodium content in food sold in China, which needs to be summarized and improved continuously. Nevertheless, more effort needs to be taken to develop approaches that would receive genuine consumer acceptance.

Information about the sodium content in foods is vital for designing a national salt reduction program. In order to investigate the sodium content in foods, 4030 pre-packaged foods on the nutrition label and 52 common artisanal foods were collected. The sodium content and the proportion of foods with low-sodium, medium-sodium and high-sodium content were further classified and analyzed. Based on the profile of sodium content in commercial food, it could help residents make informed dietary choices and contribute to achieving the goal of salt reduction in commercial food by applying practical approaches and policies.

## 2. Materials and Methods

### 2.1. Date Source and Collection

For efficiency, diversity and sample size, a simple random sampling method [24] was conducted to obtain data. Three university students from Beijing were recruited, trained and provided with a smartphone for data collection. They were required to take and store photos of the nutrition label information (front of the food package and nutrition information panel). In addition, they were required to first collect the products displayed at the middle level on the store shelves to ensure that the most common products are captured and then expanded their data collection to higher and lower levels products on the shelves. Data collection was carried out between February and August of 2021. The selection of the products was not based on the brand, price, manufacturer’s name of products, products, nor subjective thoughts of the product.

#### 2.1.1. Pre-Packaged Foods with Nutrition Labels

The sodium content data were obtained from foods with nutrition labels sold in Beijing, China, including three supermarkets (Yonghui, Wal-Mart and Wu-Mart) and an online platform (Taobao.com). Data were collected by taking pictures or given information. All the randomly picked foods were from the same category listed on the supermarkets or websites. In addition, if the same product (i.e., the same product name, nutrition information) was found in different stores, only one was included in the analysis. Foods that could not be classified, such as protein supplements and vitamins, were excluded from the study. For each food, the sodium content was recorded in Microsoft Excel 2016 (Microsoft Corporation, Redmond, WA, USA) according to the information displayed on the nutrition label.

#### 2.1.2. Determination of Sodium Content in Artisanal Foods

Artisanal foods without nutrition labels were purchased in supermarkets and canteens, including dishes, cooked food, etc. The sodium content in artisanal foods was measured as described in the literature with some modifications [25]. The soup of artisanal foods was removed, and its edible portion was shredded through a shredder. The 0.20 g sample was transferred into the digestion tank, 10 mL of concentrated nitric acid (HNO_3_) was added, and the samples were predigested at 120 °C without a cover for 40 min. Then, the sample was taken out and cooled to room temperature. After microwave digestion and acid treatment, the volume of the sample was approximately 1 mL. All samples were further diluted to a volume of 25 mL. Then, the sodium content was determined by inductively coupled plasma optical emission spectrometer (ICP-OES). The working procedures of microwave digestion and ICP-OES are shown in Supplementary Materials Tables S1 and S2.

### 2.2. Categorization of Commercial Foods

According to the classification system of the global food monitoring group and SB/T 10812-2012 of classification specification of supermarket goods [26,27], foods with nutrition labels were classified into food groups and categories in the hierarchical structure. Based on the similarity of the same food group, commercial foods were classified into 12 food groups: (i) Staple foods; (ii) Biscuits, (iii) Bread and pastry products; (iv) Sauces, dressings, spreads and dips; (v) Snack foods; (vi) Bean products; (vii) Fish, meat and egg products; (viii) Dairy products; (ix) Fruit and vegetables; (x) Confectionery products; (xi) Non-alcoholic beverages; and (xii) Artisanal foods. Detailed information on the food groups and categories is shown in Supplementary Materials Table S3.

### 2.3. Sodium Content Classification

According to the standard (GB/T 23789-2009) of low-sodium foods provided by the Chinese Government, foods with a sodium content not higher than 120 mg/100 g were classified into low-sodium foods [28]. Since there is no classification standard for high-sodium foods, the Food and Drugs (Composition and Labeling) Regulations in Hong Kong were used as the classification basis (i.e., ≥600 mg/100 g or mg/100 mL) for high-sodium foods [29]. The foods with sodium content between 120 and 600 mg/100 g or mg/100 mL were classified as medium-sodium foods.

### 2.4. Statistical Analysis

Since most liquid products are water-based, we assumed that 100 mL of liquid products is equal to 100 g to achieve a standardized report of nutritional value, which was converted into mg/100 g according to the sodium content unit on the nutrition label. Data of sodium content are presented in average, proportion and range, and the lower quartile (i.e., 25% quantile) and the upper quartile (i.e., 75% quantile) of interquartile range (IQR) is used to reflect the degree of dispersion of a distribution. In addition, the sodium content distribution of different foods was measured by calculating the proportion of low-sodium, medium-sodium and high-sodium content in each food group. Microsoft Excel 2016 (Microsoft Corporation, Redmond, WA, USA) and Origin Pro 2021 (OriginLab Corporation, Northampton, MA, USA) were used for data processing.

## 3. Results

### 3.1. Descriptive Analysis

A total of 4082 commercial foods across 12 food groups and 41 food categories were analyzed, including 4030 pre-packaged and 52 artisanal foods. The results of sodium content in food groups and food categories are shown in Table 1 and Table 2. The average sodium content of all commercial foods collected was 1018.6 mg/100 g, with a wide range of sodium content from 0 to 31,319 mg/100 g. The sodium content varied substantially among food groups. The three food groups with the highest sodium content were sauces, dressings, spreads and dips (average 6888.6 mg/100 g, IQR 3591–7986.7 mg/100 g), bean products (average 1326.1 mg/100 g, IQR 860.5–1570 mg/100 g) and fish, meat and egg products (average 1302.1 mg/100 g, IQR 900–1594 mg/100 g). In contrast, the sodium content of non-alcoholic beverages (49.7 mg/100 g, IQR 8–38.3 mg/100 g), confectionery (111.8 mg/100 g, IQR 20–135 mg/100 g), and dairy products (164.1 mg/100 g, IQR 60–200 mg/100 g) was much lower. The food categories with the largest number of products were fruit products (*n* = 257, 6.30% of all products), followed by nuts and seeds (*n* = 217, 5.32% of total), and milk (*n* = 209, 5.12% of total). Among the three food categories, fruit products have the highest average sodium content (742.9 mg/100 g), and milk has the lowest average sodium content (59.1 mg/100 g). In addition, sodium content also varied significantly within certain food categories. In the dairy products group, the average sodium content of cheese products (484.4 mg/100 g) was eight times higher than that of milk products. In the biscuits group, crackers had the highest sodium content (548 mg/100 g). In contrast, egg rolls had the lowest sodium product in this food group. Food categories also vary widely in the distribution of sodium content. For example, the sodium content of fruit products ranged from 0 to 13,440 mg/100 g, livestock meat products ranged from 11 to 6428 mg/100 g, and compound spreads ranged from 6 to 31,319 mg/100 g. In addition, meat-based dishes have higher sodium content than vegetarian-based dishes in the collected products.

### 3.2. Comparison of Low-, Medium-, and High-Sodium Foods

**Figure 1:**
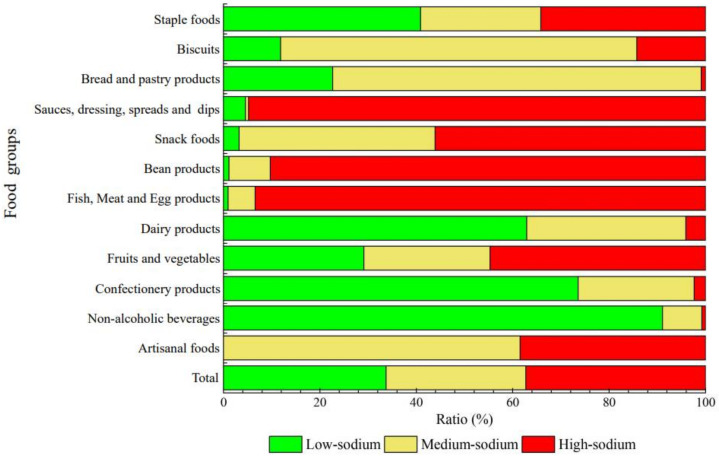
The proportion of low-, medium-, and high-sodium content in food groups.

**Figure 2:**
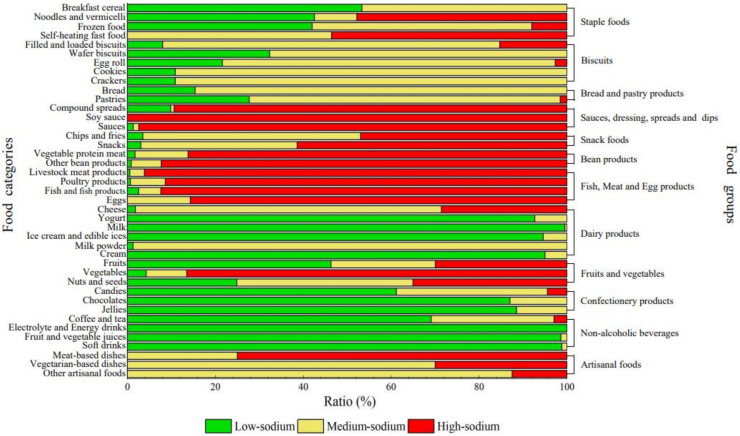
The proportion of low-, medium- and high-sodium content in food categories.

According to the standard of low-, medium-, and high-sodium foods, 33.72% of the collected products met the low-sodium standard, and 37.30% of them met the high-sodium standard. The proportions of high-, medium- and low-sodium in different food groups and food categories are shown in Figure 1 and Figure 2. It can be seen from Figure 1 that the proportion of low-sodium, medium-sodium and high-sodium products varied greatly among food groups. Groups with the highest proportion of high-sodium products were sauces, dressings, spreads and dips (94.82%), followed by fish, meat and egg products (93.43%), and bean products (90.29%). In contrast, more than 60% of the products of non-alcoholic beverages, confectionery products and dairy products met the standard of low-sodium food. As shown in Figure 2, most liquid-based food categories are mainly low-sodium food. For example, in the food group of dairy products, milk (100.00%) and yogurt (97.00%) basically belong to low-sodium foods, while milk powder basically belongs to medium-sodium foods (98.75%). In addition, there is no low-sodium product among the 52 artisanal foods. Medium-sodium food dominated the vegetarian-based artisanal foods with a proportion of 70.00%. Meat-based artisanal foods were mainly high-sodium foods (75.00%), which is twice as much as that of vegetarian-based artisanal foods.

### 3.3. Sodium Content in Different Brands of Soy Sauce and Bread Products

Due to the different dietetic cultures, the main sources of sodium intake vary greatly from country to country. Western countries, such as European countries and the United States, have similar dietary backgrounds, under which bread and other baked products are the main sources of sodium intake. As a less industrialized country, common salt and sauces used in daily cooking are the key factors of sodium consumption in China [30]. The sodium content of products from different brands also varied greatly within a certain category. Thus, statistical analysis was conducted on soy sauce and bread products with different brands. Ninety-three soy sauce products from 11 brands (Supplementary Materials Table S4) were analyzed, in which brand 3 provided 14 soy sauce products. The sodium content of the 11 brands of soy sauce is shown in Table 3. The average sodium content of soy sauces from different brands ranged from 4441.4 mg/100 g (Brand 4) to 7672.0 mg/100 g (Brand 7). The maximum difference in the average sodium content is more than 3000 mg/100 g. There were also significant differences between individual products. The soy sauce with the highest sodium content was twice as much as the product with the lowest sodium content. In addition, all the soy sauce products belong to high-sodium food. As one of the most popular foods globally, bread has a variety of brands and product types. Seventy-one products from 13 brands of bread were analyzed, and Brand 15 provided the most products (11 bread products). The sodium content of 13 different bread brands is shown in Table 4. The difference in average sodium content was more than 200.0 mg/100 g among the 13 brands, from 134.2 mg/100 g (brand 19) to 338.0 mg/100 g (brand 20). Among the bread products, the maximum difference in the average sodium content is more than 200 mg/100 g. In addition, the bread product with the highest sodium content was eight times as much as the product with the lowest sodium content.

### 3.4. Comparison between Non-Salt-Reduced and Salt-Reduced Products

**Figure 3:**
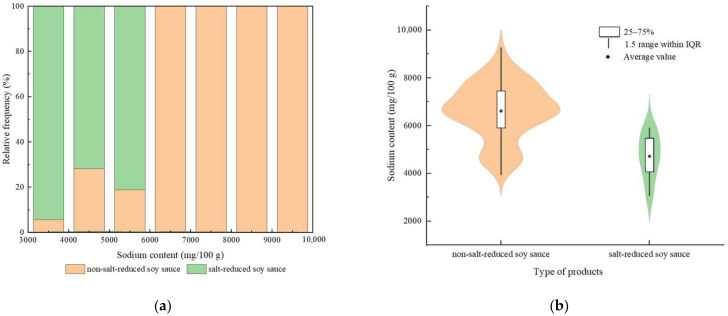
The sodium content of non-salt-reduced soy sauce and salt-reduced soy sauce. (**a**) The relative proportion of different sodium content ranges. (**b**) Violin plots of sodium content. Note: The “IQR” means interquartile range.

Sodium-reduced products attract more attention due to the demand for a healthy diet. Food products, advertised for reduction to attract consumers, have appeared in the market in recent years. In this research, the salt-reduced products collected in commercial foods are mainly concentrated in sauces, dressings, springs and dips, among which the most salt-reduced products were soy sauces. Taking soy sauce products as an example, 16 different salt-reduced soy sauces were identified, which occupied 16% of soy sauce products. It can be seen from Figure 3 that the sodium content of salt-reduced soy sauce products was mainly concentrated in the region of 3000–6000 mg/100 g, while the non-salt-reduced soy sauce products were mainly concentrated in the region of 6000–10,000 mg/100 g. The average sodium content of non-salt-reduced soy sauce was 6666.39 mg/100 g, and the average sodium content of salt-reduced soy sauce was 4643.56 mg/100 g. Although soy sauce products belong to high-sodium food, salt-reduced soy sauce cut down the sodium content by nearly 2000 mg/100 g compared with non-salt-reduced products. The sodium content of salt-reduced soy sauce decreased significantly.

## 4. Discussion

Sodium is an essential nutrient for the human body. However, excessive sodium intake leads to the increasing prevalence of diseases [31]. With the increase in the consumption of pre-packaged foods, sodium in pre-packaged foods has gradually become an important source of dietary sodium. Therefore, monitoring sodium content is crucial to evaluate the success and deficiency of efforts made by the food industry to reduce the sodium content in foods [32].

By comparing the sodium content of 12 food groups, there were significant differences between food groups. The sodium content of sauces, dressings, spreads and dips is the highest, and these products basically belong to high-sodium foods. It is consistent with previous studies on the sodium content of foods in UK, India and other countries [33,34]. However, the sodium content of these categories in China was significantly higher than that in other countries. Sauces, dressings, spreads and dips are mainly used as auxiliary materials in making dishes, and they are not eaten directly. In addition, more than 90% of the meat products belong to high-sodium foods, and it is also a primary source of sodium intake. Sodium salt plays a key role in the preservation of meat products, and it helps to improve the sensory quality [35]. Among all dairy products, cheese has the highest average sodium content (484.41 mg/100 g), while the average sodium content of other categories in dairy products was less than 20% of the cheese. The addition of salt could inhibit the formation of biogenic amines by bacteria, which is necessary for cheese fermentation [36]. Consistent with previous studies, most of these low-sodium foods come from categories of milk, sugar, and beverages. Significant differences in the sodium content of different food groups and categories were observed, and it might be a good way to set different standards for low-, medium- and high-sodium content in a wide range of food categories to achieve the goal of sodium reduction.

Due to the change in lifestyle and the development of food industrialization, China has become one of the largest consumer markets of pre-packaged foods. It is found that even for the same food category, there are great differences in salt content among different brands. Thus, the data of soy sauce (11 brands) and bread (13 brands) were further analyzed, which represents one of the main sodium sources in the Chinese and Western diets, respectively. Basically, soy sauce was classified as a high-sodium food, while most of the bread products were in the range of medium-sodium food. The soy sauce brand and bread brand with the highest average sodium content was 2.52 times and 1.73 times as much as the brands with the lowest average sodium content. Bread seems to have a lower level of sodium content compared with soy sauce, but it is the staple food and one of the main sources of salt in the Western diet with a large daily intake. The voluntary salt reduction program implemented in the UK through reformulation has successfully reduced the sodium content of many foods, including bread [37]. For foods with a wide range of sodium content, it is technically feasible to reduce the sodium content by reformulation [38]. Although the continuous emergence of low-sodium foods reflects that they have been reconstituted worldwide, there are limitations to reducing the sodium content in pre-packaged foods by reformulating. For example, reducing the sodium content may shorten the shelf life of meat and meat products [36]. In addition, the lower consumer acceptability of low-sodium foods is another reason why food manufacturers are reluctant regarding reformulation [29]. However, studies showed that most consumers agree with the Government to restrict food manufacturers from adding excessive salt to food [39]. Therefore, the Government should consider the reasonable objectives of food manufacturers when setting any goals. At present, the concentration of sodium in food products could be reduced by replacing sodium with substitutes, redesigning the food structure, and changing the physical properties of salt [40]. It is also an alternative way to reduce NaCl concentration without sacrificing the palatability of food by using flavor enhancers, such as monosodium glutamate (MSG), disodium 5’-inosinate (IMP), and non-sodium umami agents [41,42,43]. The reduction in sodium content in soy sauce was helpful in reducing the sodium content in artisanal foods, thus reducing the daily sodium intake. There are salt reduction products on the market, which also reflects the efforts made by the Government and food manufacturers in food salt reduction. However, there are few types of salt-reduced products, and these are mainly concentrated in sauces, dressings, springs and dips. 

Considering the recommendation of sodium intake for adults from the WHO, the intake limitations (per person per day) of different food groups were calculated based on the IQR, average and max sodium content (Supplementary Materials Table S5). As diverse dietary patterns contribute to the daily sodium intake of residents, the actual intake of a certain food group should be lower than the limitation. The food group with the highest sodium content was sauces, dressings, spreads and dips, and the daily intake of these products should not exceed 29.03 g on average. In addition, the high-sodium proportion of bean products and fish, meat and egg products was more than 90%, and the intake limitations of these two food groups were 150.82 g and 153.60 g per day. It is generally agreed that saltiness is not the dominant flavor of staple foods and fruit products, but it is noteworthy that some of these products had a high-sodium content. For example, fine dried noodles and preserves have a sodium content up to 3616 mg/100 g and 13,440 mg/100 g, respectively. Intake of 55.31 g fine dried noodles or 14.88 g preserves could reach the maximum daily sodium intake from the WHO (2000 mg per person per day). The intake limitation range based on the IQR of different food groups indicated that less sodium intake choices are available, and the nutritional data or labels on the package will help consumers make healthier food choices. The Chinese Food Guide Pagoda, derived from the Chinese Dietary Guidelines (2022), was an illustration of the recommended intake of various foods. The pagoda was divided into five layers, including grains and potatoes, vegetables and fruits, animal foods, dairy products, and cooking oil and salt. Based on each layer of the pagoda, the food categories in this study were roughly classified and combined. According to the approximate proportion of home cooking and pre-packaged food intake, 20% of recommended daily intake in the pagoda was considered for providing combinations of pre-packaged foods (Supplementary Materials Table S6), which could help consumers make reasonable dietary choices in the Chinese supermarkets. The daily sodium intake of combination 1–4 was less than 1000 mg/day while combination 5–8 ranged from 1000 to 2000 mg/day, and combination 9–12 was higher than 2000 mg/day. There are clear standards for low-sodium food in pre-packaged food in China. The standard further divides low-sodium food into ordinary low-sodium food and very low-sodium food, and it should be marked on the nutrition label. However, the standard of high-sodium food is not clearly pointed out. Some countries have been using “traffic light labeling” to distinguish sodium content in different colors, in which high-sodium foods are marked by red and low-sodium foods are marked by green [44]. Although there is evidence that “traffic light labeling” may help consumers make healthier choices and improve dietary intake, it is not implemented in China at present. Therefore, the Government and policymakers could consider adopting color marking to alert consumers about high-/low-sodium content on the food package when revising nutrition labeling regulations. It is a practical action and cost-effective strategy to reduce the sodium for governments and individuals. Meanwhile, by indicating the high-, medium- and low-sodium content, nutrition labels could efficiently help consumers choose appropriate food and urge manufacturers to reduce the sodium content.

The nutrition label on pre-packaged food is one of the convenient ways to obtain sodium content. In addition, the nutrition label is considered to be an effective way to inform consumers of food nutrients and may affect their eating habits [45]. Among the 52 artisanal foods, none of them are low-sodium foods, and they are mainly distributed in the range of medium-sodium food. The sodium in artisanal foods is mainly derived from the sodium contained in the ingredients and salty seasonings, such as salt and monosodium glutamate, which help to improve the flavor and extend the shelf life [46]. Compared with Western countries, most of the dietary sodium in China is introduced from salt when cooking at home and on the table. Therefore, the sodium content in artisanal foods is worthy of attention. At present, there is no requirement for artisanal foods to list the content of nutrients. There is no direct access to the sodium content of artisanal foods for consumers. The artisanal foods are mainly from manual production by the supermarket, small businesses or family groups [47]. The salt content in artisanal food is greatly affected by the food handlers, and there are differences in sodium content for different batches of products. Considering the demand for healthy diets, a reasonable range of sodium content could be regulated and displayed in the sales window according to different types of artisanal food.

Online shopping has become a new way of consumption, and it is also an important way to buy pre-packaged foods nowadays [48]. The survey data came from commercial foods, including many foods sold only on online platforms. The products with the same brand, product name and nutritional components are classified into one kind to avoid data overlap as much as possible. However, the current research still has some limitations. First, the sodium content was mainly obtained from the nutrition labels. Although this is the most economical way to obtain reliable nutritional information, the authenticity of sodium content on nutrition labels could be further verified by chemical analysis. In addition, the sodium content of 4082 products was analyzed in this study; however, the comprehensiveness and accuracy of the data could be improved by increasing the sample size and instrumental analysis. To provide sufficient support for scientific salt reduction in the food industry, consumer consumption of different categories of food needs to be taken into account in future research.

## 5. Conclusions

The sodium content of 4030 pre-packaged foods and 52 artisanal foods was involved in this study. The average sodium content of all the collected commercial foods was 1018.6 mg/100 g, with a wide range from 0 to 31,319 mg/100 g. The food groups of sauces, dressings, springs and dips showed the highest average sodium content (6888.6 mg/100 g), while the sodium content of non-alcoholic beverages was much lower. Among all commercial foods, more than one-third of products meet the standard of high-sodium food; besides, none of the 52 artisanal foods belongs to low-sodium food. Only 16 sodium-reduced soy sauce products were collected in this survey, and their sodium content was reduced by 2022.8 mg/100 g on average when compared with non-salt-reduced soy sauce. Significant differences in the sodium content of different food groups, categories and brands were observed, which demonstrated the considerable potential for food reformulation to reduce the sodium content. Practical action and cost-effective strategies should be adopted to reduce sodium for governments and individuals, such as setting feasible standards for low-, medium- and high-sodium food for different food categories and color marking to alert the sodium content on food packages.

## Figures and Tables

**Table 1:** The sodium content by group for commercial foods.

Food Group	*n*	Proportion	Sodium Content (mg/100 g)		
		(%)	IQR	Range	Average
Staple foods	345	8.45	50.0–659.0	0–4150.0	517.9
Biscuits	295	7.23	197.0–466.0	14.0–935.0	343.1
Bread and pastry products	221	5.41	149.0–311.0	10.0–782.0	236.5
Sauces, dressings, spreads and dips	309	7.57	359.01–7986.7	6.0–31,319.0	6888.6
Snack foods	312	7.64	470.0–803.2	45.0–1786.0	644.8
Bean products	175	4.29	860.5–1570.0	11.0–6428.0	1326.1
Fish, meat and egg products	426	10.44	900.0–1594.0	11.0–6428.0	1302.1
Dairy products	779	19.08	60.0–200.0	20.0–1490.0	164.1
Fruits and vegetables	615	15.07	71.0–1152.5	0–13,440.0	839.1
Confectionery products	295	7.23	20.0–135.0	0–1300.0	111.8
Non-alcoholic beverages	258	6.32	8.0–38.3	0–990.9	49.7
Artisanal foods	52	1.27	341.9–477.5	169.3–3201.0	668.6
Total	4082	100	64.0–917.3	0–31,319.0	1023.1

Note: The “*n*” means the number of products. The “IQR” means interquartile range.

**Table 2:** The sodium labels and content by category for commercial foods.

Food Group	Food Category	*n*	Proportion	Sodium Content (mg/100 g)		
			(%)	IQR	Range	Average
Staple foods	Breakfast cereal	60	1.49	35.8–168.5	0–561.0	122.7
	Noodles and vermicelli	207	5.14	50.0–800.0	0–3616.0	627.2
	Frozen food	50	1.24	36.3–380.5	0–2420.0	288.7
	Self-heating fast food	28	0.69	360.5–1376.4	283.0–4150.0	966.3
Biscuits	Filled and loaded biscuits	137	3.4	255.6–499.0	14.0–935.0	387.3
	Wafer biscuits	34	0.84	114.0–219.9	80.0–542.0	196.7
	Egg roll	37	0.92	129.0–240.0	32.0–673.0	194.3
	Cookies	46	1.14	160.5–333.3	24.0–536.0	245.7
	Crackers	41	1.02	455.0–645.0	176.0–734.0	548
Bread and pastry products	Bread	91	2.26	191.0–273.0	50.0–548.0	239.6
	Pastries	134	3.33	100.5–332.3	10.0–782.0	234.3
Sauces, dressings, spreads and dips	Compound spreads	132	3.28	4377.5–13,632.3	6.0–31,319.0	9584.1
	Soy sauce	100	2.48	5668.3–7361.7	3060–9273.3	6397.9
	Sauces	77	1.91	1570.0–4300.0	68.0–8200.0	2905.2
Snack foods	chips and fries	115	2.85	393.3–722.5	83.3–1123.0	561.9
	Snacks	197	4.89	494.0–850.0	45.0–1786.0	693.2
Bean products	Vegetable protein meat	58	1.44	802.8–1196.5	60.0–2771.0	1071.7
	Other bean products	117	2.9	863.0–1500.0	28.0–3300.0	1322.7
Fish, meat and egg products	Livestock meat products	182	4.52	860.5–1570.0	11.0–6428.0	1326.1
	Poultry products	151	3.75	924.5–1661.5	106.0–3318.0	1329.7
	Fish and fish products	79	1.96	906.0–1626.0	35.0–2596.0	1238.6
	Eggs	14	0.35	766.8–1216.8	35.0–2596.0	1048.7
Dairy products	Cheese	112	2.78	280.0–662.5	30.0–1490.0	484.4
	Yogurt	204	5.06	53.0–70.0	20.0–208.0	68.9
	Milk	209	5.19	50.0–65.0	35.0–213.0	59.1
	Ice cream and edible ices	74	1.84	44.6–80.0	20.0–169.0	66.3
	Milk powder	160	3.97	150.0–340.0	111.4–550.0	256.2
	Cream	20	0.5	30.0–66.5	20.0–169.0	63.3
Fruits and vegetables	Fruits	257	6.38	35.0–923.0	0–13,440.0	742.9
	Vegetables	141	3.5	746.0–1820.0	0–12,300.0	1525
	Nuts and seeds	217	5.38	136.0–794.0	0–1578.0	507.4
Confectionery products	Candies	157	3.9	24.0–213.0	0–1300.0	154.8
	Chocolates	77	1.91	18.0–88.0	0–392.0	68.2
	Jellies	61	1.51	30.0–70.0	9.0–145.0	56.1
Non-alcoholic beverages	Coffee and tea	68	1.69	20.0–182.2	0–990.9	130.1
	Electrolyte and Energy drinks	35	0.87	14.5–47.0	0–118.0	34.1
	Fruit and vegetable juices	71	1.76	0–29.0	0–123.0	22
	Soft drinks	84	2.08	6.0–16.0	0–139.0	14.6
Artisanal foods	meat-based dishes	16	0.39	595.2–798.5	365.5–1686.0	829.9
	Vegetarian-based dishes	20	0.49	325.2–710.4	222.2–3201.0	766
	other artisanal foods	16	0.39	235.9–346.0	169.3–802.2	385.5

Note: The “*n*” means the number of products. The “IQR” means interquartile range.

**Table 3:** The sodium content in 11 different brands of soy sauce.

Brand	*n*	Average Sodium Content (mg/100 g)	SD (mg/100 g)	Range of Sodium Content (mg/100 g)
1	6	6446.7	587.3	5810–7200
2	11	6829.1	1044.3	4813.3–7860
3	14	6498.1	1023.6	4600–7986.7
4	13	4626.4	1040.7	3060–6666.7
5	11	7481.2	1193.6	5000–9273.3
6	6	5040.0	793.0	4520–6620
7	10	7672.0	818.0	5633.3–8440
8	6	5850.0	1055.7	3753.3–6500
9	5	6881.3	1133.9	5906.7–8640
10	3	6664.4	348.4	6466.7–7066.7
11	8	6648.3	158.7	6533.3–6840

Note: The “*n*” means number of products, and the “SD” means standard deviation.

**Table 4:** The sodium content in 11 different brands of bread.

Brand	*n*	Average Sodium Content (mg/100 g)	SD (mg/100 g)	Range of Sodium Content (mg/100 g)
12	4	302.8	164.5	208–508
13	3	238.7	11.9	225–246
14	4	303.0	175.6	68–492
15	11	301.1	73.4	243–466
16	4	256.0	19.8	239–276
17	6	244.0	93.4	100–393
18	9	205.0	72.8	97–325
19	5	134.2	87.3	70–288
20	5	222.6	85.2	175–372
21	4	249.3	14.5	230–265
22	3	237.3	50.2	209–295

Note: The “*n*” means number of products, and the “SD” means standard deviation.

## Data Availability

The data presented in this study are available on request from the corresponding author.

## Supplementary Materials

The following supporting information can be downloaded at: https://www.mdpi.com/article/10.3390/nu14142908/s1, Table S1: Microwave digestion program; Table S2: ICP-OES measurement conditions.; Table S3: Food groups and categories of commercial foods; Table S4: Detailed information on different brands; Table S5: Daily intake limitation of food groups; Table S6: Combinations of dietary choices on pre-packaged food.
