# Supplementary material for: Analysis of Sodium Content in 4082 Kinds of Commercial Foods in China
Source: Nutrients. 2022 Jul 15;14(14):2908. doi: 10.3390/nu14142908 (PMC9322708; doi:10.3390/nu14142908)
Supplement: Supplementary file 1 [file nutrients-14-02908-s001.zip › nutrients-1802133-supplementary.pdf]

Table S1. Microwave digestion program.

Step	Power/W	Time/min	Temperature/℃
1	1000	10	150
2	1000	15	180

Table S2. ICP-OES measurement conditions.

Parameter and accessories		Value
Gas type and power		Argon 450 ± 10 kPa
Exposure time		30 s
Solvent rinse		30 s
Plasma view		axial view
Gas flow rate	Plasma gas flow rate	10 L/min
	Auxiliary gas flow rate	0.6 L/min
	Carrier gas flow rate	0.7 L/min
Peristaltic pump	Solvent rinse	30 s
	Sample rinse	30 s
Instrument status	CDC temperature	＜−15 ℃
	degree of vacuum	＜10 Pa
Analysis wavelength	Sodium	588.995 nm

Note: The "ICP-OES" means "inductively coupled plasma—optical emission spectrometry", the "CCD" means "charge coupled device".

Table S3. Food groups and categories of commercial foods.

Food Group	Food Category
Staple foods	Breakfast cereal<br>Noodles and vermicelli<br>Frozen food<br>Self-heating fast food
Biscuits	Filled and loaded biscuits<br>Wafer biscuits<br>Egg roll<br>Cookies<br>Crackers
Bread and pastry products	Bread<br>Pastries
Sauces, dressing, spreads and dips	Compound spreads<br>Soy sauce<br>Sauces
Snack foods	chips and fries<br>Snacks
Bean products	Vegetable protein meat<br>Other bean products
Fish, Meat and Egg products	Livestock meat products<br>Poultry products<br>Fish and fish products<br>Eggs
Dairy products	Cheese<br>Yogurt<br>Milk<br>Ice cream and edible ices<br>Milk powder<br>Cream
Fruits and vegetables	Fruits<br>Vegetables<br>Nuts and seeds
Confectionery products	Candies<br>Chocolates<br>Jellies
Non-alcoholic beverages	Coffee and tea<br>Electrolyte and Energy drinks<br>Fruit and vegetable juices<br>Soft drinks
Artisanal foods	meat-based dishes<br>Vegetarian-based dishes<br>other artisanal foods

Table S4. Detailed information on different brands.

Brand	Name	Producing City	Brand	Name	Producing City
1	Qian He	Meishan, China	13	OBERAI	Quanzhou, China
2	Lee Kum Kee	Zhuhai, China	14	Bestore	Jinjiang, China
3	HA DAY	Foshan, China	15	Three Squirrels	Wuhu, China
4	Shinho	Yantai, China	16	honyifood	Quanzhou, China
5	Chu Bang	Zhongshan, China	17	BIBIZAN	Zhangzhou, China
6	Luhua	Yantai, China	18	Toly Bread	Chengdu, China
7	Donggu	Jiangmen, China	19	Calleton	Zhangzhou, China
8	Kikkoman	Suzhou, China	20	Qinianwuji	Wuhan, China
9	Jia Jia	Changsha, China	21	She li	Zhengzhou, China
10	Xue Tai Feng	Wuxi, China	22	Panpan food	Chuzhou, China
11	Huang Hua Yuan	Chongqing, China	23	Xiaobai-home	Zhangzhou, China
12	Be & Cheery	Dongguan, China	24	Qian Si	Quanzhou, China

Table S5. Daily intake limitation of food groups.

Food group	The daily limitation (g/per person per day)		
	Based on average sodium content	Based on Max sodium content	Based on IQR
Staple foods	386.17	48.19	303.46–4000.00
Biscuits	582.92	213.90	429.18–1015.23
Bread and pastry products	845.67	255.75	643.09–1342.28
Sauces, dressing, spreads and dips	29.03	6.39	25.04–557.09
Snack foods	310.17	111.98	249.00–425.53
Bean products	150.82	31.11	127.39–232.42
Fish, Meat and Egg products	153.60	31.11	125.47–222.22
Dairy products	1218.77	134.23	1000.00–3333.33
Fruits and vegetables	238.35	14.88	175.54–2816.90
Confectionery products	1788.91	153.85	1481.48–10,000.00
Non-alcoholic beverages	4024.14	201.84	5221.93–25,000.00
Artisanal foods	299.13	62.48	418.85–584.97
Total	195.48	6.39	218.03–3125.00

Note: The "Max" means "maximum value", the "IQR" means "interquartile range".

Table S6. Combinations of dietary choices on pre-packaged food.

Food combination	Food Category	Average sodium content (mg/100 g)	Recommended intake (g/day)	Sodium intake (mg/day)
1	Milk	59.1	80	47.28
	Eggs	1048.7	32	335.584
	Fruits	742.9	55	408.595
	Breakfast cereal	122.7	50	61.35
	Total			852.809
2	Milk	59.1	40	23.64
	Yogurt	68.9	40	27.56
	Poultry products	1329.7	16	212.752
	Eggs	1048.7	16	167.792
	Fruits	742.9	55	408.595
	Bread	239.6	50	119.8
	Total			960.139
3	Yogurt	68.9	80	55.12
	Livestock meat products	1326.1	16	212.176
	Eggs	1048.7	16	167.792
	Nuts and seeds	507.4	6	30.444
	Fruits	742.9	55	408.595
	Bread	239.6	25	59.9
	Pastries	234.3	25	58.575
	Total			992.602
4	Milk	59.1	40	23.64
	Yogurt	68.9	40	27.56
	Poultry products	1329.7	16	212.752
	Livestock meat products	1326.1	16	212.176
	Fruits	742.9	55	408.595
	Wafer biscuits	196.7	25	49.175
	Bread	239.6	25	59.9
	Total			993.798
5	Milk	59.1	40	23.64
	Yogurt	68.9	40	27.56
	Livestock meat products	1326.1	32	424.352
	Fruits	742.9	55	408.595
	Frozen food	288.7	50	144.35
	Total			1028.497
6	Cheese	484.4	40	193.76
	Yogurt	68.9	40	27.56
	Livestock meat products	1326.1	8	106.088
	Poultry products	1329.7	8	106.376
	Fish and fish products	1238.6	8	99.088
	Eggs	1048.7	8	83.896
	Fruits	742.9	55	408.595
	Bread	239.6	25	59.9
	Noodles and vermicelli	627.2	25	156.8
	Total			1242.063

7	Milk	59.1	80	47.28
	Nuts and seeds	507.4	6	30.444
	Poultry products	1329.7	16	212.752
	Fish and fish products	1238.6	16	198.176
	Vegetables	1525	80	1220
	Pastries	234.3	25	58.575
	Frozen food	288.7	25	72.175
	Total			1839.402
8	Milk	59.1	80	47.28
	Fish and fish products	1238.6	32	396.352
	Vegetables	1525	80	1220
	Noodles and vermicelli	627.2	50	313.6
	Total			1977.232
9	Milk	59.1	80	47.28
	Poultry products	1329.7	16	212.752
	Eggs	1048.7	16	167.792
	Vegetables	1525	80	1220
	Filled and loaded biscuits	387.3	25	96.825
	Vegetable protein meat	1071.7	25	267.925
	Total			2012.574
10	Milk	59.1	40	23.64
	Cheese	484.4	40	193.76
	Nuts and seeds	507.4	6	30.444
	Livestock meat products	1326.1	16	212.176
	Poultry products	1329.7	16	212.752
	Vegetables	1525	80	1220
	Bread	239.6	25	59.9
	Cookies	245.7	25	61.425
	Total			2014.097
11	Cheese	484.4	80	387.52
	Livestock meat products	1326.1	16	212.176
	Eggs	1048.7	16	167.792
	Fruits	742.9	55	408.595
	Vegetables	1525	80	1220
	Noodles and vermicelli	627.2	25	156.8
	Crackers	548	25	137
	Total			2689.883
12	Cheese	484.4	80	387.52
	Nuts and seeds	507.4	6	30.444
	Livestock meat products	1326.1	32	424.352
	Fruits	742.9	55	408.595
	Vegetables	1525	80	1220
	Noodles and vermicelli	627.2	50	313.6
	Total			2784.511
